# PROGpedia: Collection of source-code submitted to introductory programming assignments

**DOI:** 10.1016/j.dib.2023.108887

**Published:** 2023-01-10

**Authors:** José Carlos Paiva, José Paulo Leal, Álvaro Figueira

**Affiliations:** a Department of Computer Science from the Faculty of Sciences, University of Porto, Porto, Portugal; b CRACS - INESC TEC, Porto, Portugal

**Keywords:** Programming learning, Source code, Semantic representation, Automated assessment, Abstract Syntax Tree, Control-flow, Data-flow, Code Property Graph, AST, Abstract Syntax Tree, CLI, Command-Line Interface, CPG, Code Property Graph, CS, Computer Science, CSV, Comma-Separated Values

## Abstract

Learning how to program is a difficult task. To acquire the required skills, novice programmers must solve a broad range of programming activities, always supported with timely, rich, and accurate feedback. Automated assessment tools play a major role in fulfilling these needs, being a common presence in introductory programming courses. As programming exercises are not easy to produce and those loaded into these tools must adhere to specific format requirements, teachers often opt for reusing them for several years. Therefore, most automated assessment tools, particularly Mooshak, store hundreds of submissions to the same programming exercises, as these need to be kept after automatically processed for possible subsequent manual revision. Our dataset consists of the submissions to 16 programming exercises in Mooshak proposed in multiple years within the 2003–2020 timespan to undergraduate Computer Science students at the Faculty of Sciences from the University of Porto. In particular, we extract their code property graphs and store them as CSV files. The analysis of this data can enable, for instance, the generation of more concise and personalized feedback based on similar accepted submissions in the past, the identification of different strategies to solve a problem, the understanding of a student's thinking process, among many other findings.

**Specifications Table**

**Table:** 

Subject:	Computer Science > Human-Computer Interaction
Specific subject area:	Analysis of the code property graphs from the source code submitted by undergraduate CS students into automated assessment tools.
Type of data:	File system directories MarkDown files CSV files Java, C, C++, and Python text files
How the data were acquired:	Data were collected from Mooshak instances, which have supported undergraduate Computer Science students at the Faculty of Sciences from the University of Porto for multiple years within the 2003–2020 timespan. Mooshak submissions include source code, binaries, and assessment results. This data has been processed automatically to (1) extract the code property graph into CSV (nodes' and edges' files) and (2) organize these files in a three-level directory tree, in which: the first level is the problem folder; the second is the result of the submission; and the third is the pair submitter-order of attempt. The CSV files are inside the respective third-level directory.
Data format:	Raw Analyzed Filtered
Description of data collection:	The source code must have been submitted to one of the selected programming exercises and evaluated by Mooshak successfully (i.e., assigned a classification). We required a minimum of 100 submissions of distinct participants to include the programming exercise. Data is completely anonymized, as no reference to the participant is kept. Only a newly auto-increment ID per programming assignment is used to distinguish different submitters.
Data source location:	Institution: Faculty of Sciences from the University of Porto City/Town/Region: Porto Country: Portugal
Data accessibility:	Repository name: Zenodo Data identification number: 10.5281/zenodo.7449056 Direct URL to data: http://doi.org/10.5281/zenodo.7449056

## Value of the Data

• These data are useful because they concentrate on the semantic and syntactic value of the source code of all attempts to solve a set of programming problems from previous students. Given a new attempt, this allows us to: (1) match it with the group of solutions following the same/similar strategy; (2) identify the closest correct solution; and (3) draw the differences that transform the current source code into a correct one.
• The data primarily benefit researchers in the area of automated assessment of programming assignments, particularly, for improving automatically generated feedback, which is one of the hottest topics [1]. Moreover, research in Learning Analytics for Computer Science education can also obtain great value from these data (e.g., how a student progresses towards a correct solution).
• These data can be used to research problems such as automatic feedback generation for programming assignments, understanding students’ program development process, and assessing the relationship between the different problem-solving strategies and difficulty in achieving a correct solution.

## 1 Objective

Practice is the key to programming learning. Every year undergraduate CS students submit hundreds of source code files to the chosen automated assessment tool. Beyond the initial correctness assessment and storage for a possible manual verification, all this source code is typically just put aside as useless. At the same time, feedback generated by these tools is still far from pedagogical effectiveness [1]. This dataset aims to reason over past submission data to produce feedback on how to rework the current erroneous attempt to make it correct. In particular, given a new program, the idea is to (1) select the closest known working solution, (2) draw the differences between both extracted graph representations, and (3) use them to generate concise and incremental feedback messages. To this end, having a representation such as the code property graph [2] that combines the syntactic and semantic values of a program is important. On the one hand, measuring semantic similarity is far less complex than syntactical. On the other hand, making the program semantically identical to a correct solution will most likely not fix it.

## 2 Data Description

The dataset [3] consists of the submissions to 16 programming assignments delivered to undergraduate Computer Science (CS) students on intermittent years within the 2003–2020 through Mooshak [4]. Mooshak assigns twelve possible classifications to a program. For the purpose of this dataset, we consider only four classifications which may correspond to multiple Mooshak classifications, namely: Accepted, which is assigned to fully correct programs as well as programs with minor presentation errors in output; Wrong Answer, which labels programs with incorrect output; Compile Error, that specifies programs that do not compile; and Runtime Error, which classifies programs that error out during execution for any reason. Compilation Errors typically prevent the extraction of a source code representation and, thus, were excluded from the dataset. 44 programming assignments were excluded for lacking the amount of participants considered as minimum to be part of the dataset, 100 (i.e., a total of 60 were initially considered). Table 1 summarizes the dataset according to the submission count, participant count, and classification by problem. Note: participants are not matched across distinct problems.

**Table 1:** Statistics of the dataset before processing.

			Classification		
ID	Submission Count	Participant Count	ACCEPTED	WRONG ANSWER	RUNTIME ERROR
00000006	546	226	211	141	194
00000016	263	121	156	72	35
00000018	349	102	71	65	213
00000019	663	205	244	303	116
00000021	937	176	156	439	342
00000022	372	120	119	164	89
00000023	405	123	128	136	141
00000034	839	199	403	65	371
00000035	833	258	241	249	343
00000039	1027	237	256	297	474
00000042	671	210	223	281	167
00000043	576	192	288	250	38
00000045	652	212	224	313	115
00000048	367	160	191	112	64
00000053	372	133	197	68	107
00000056	245	100	108	106	31
**Total**	**9117**	**2774**	**3216**	**3061**	**2840**

Each submission consists of:

• a TCL file, named .data.tcl, with metadata about the submission such as the owner, programming language, problem, and creation timestamp, and the obtained classification through the typical input-output test-based assessment;
• the source code file, which is either .c, .cpp, .java, or .py depending on the programming language used C, C++, Java, or Python respectively.

Submissions’ files are organized in a two-level directory tree, where the first level corresponds to the problem ID and the second level is the submission folder, named by its ID.

After processing, a three-level directory tree is generated, in which: the first level is the problem folder; the second is the classification of the submission; and the third is the pair submitter-order of attempt (submitter is an auto-generated index that updates both per problem and participant). This last level holds two files:

• a CSV file, named as *.dsc.csv, containing information about the code property graph's nodes. Each row describes a node, including the ID, type, token, location in the source code, and actual source code snippet;
• a CSV file, named like *.cpg.csv, describing the edges of the code property graph, including the source and target nodes, type, index (i.e., defines the order for edges of the same type between the same nodes), branch flag (i.e., for branches, indicates whether it is the truth case - true - or the else case - false), and name (i.e., for passing input data into a function through its named arguments).

Furthermore, the problems’ folders contain a Markdown file, statement.md, presenting the challenge to solve, in English, as well as one or more sample test cases. The root of the dataset archive contains a Markdown file, data.md, describing the names, definitions, and attributes of the elements of this dataset (i.e., the data dictionary).

## 3 Experimental Design, Materials and Methods

The designed data collection process starts with a manual file system copy of the submission directories of the old Mooshak instances’ backups to a common directory, which resembles that described in the Data section (i.e., before processing). A python script, specifically developed to prepare this dataset, takes the path to the root of this directory to process it.

The processing phase consists of iterating through each programming assignment and each submission inside these, and for each of them

1. parse metadata and analysis result from .data.tcl file of the submission;
2. create adequate missing branches in the directory tree within the output path for this submission (i.e., the folder named as the problem ID, a folder inside with the classification of the submission, and another inside named with the pair submitter-order of attempt separated with underscore);
3. extract the code property graph from the source code and export as CSV into the respective directory.

For the latter, an existing Kotlin library [5], designed to extract the code property graph (CPG) out of source code in C/C++ (C17) and Java (Java 13), with experimental support for several other programming languages such as Python, Golang, and TypeScript, has been adapted. The changes were twofold: (1) develop functionality to export the representations into comma-separated value (CSV) format and (2) integrate the CLI in a Docker container. The container is managed from the main Python script, which completely builds the dataset.

Problem statements, present in each problem folder, were manually translated to English from their original Portuguese version. These statements are copied using the script to the respective problem folder.

## Ethics Statements

Participation in this dataset is fully anonymized and voluntary. Participants have been informed upfront that the data they produce and submit would be automatically processed by static analysis tools.

## CRediT authorship contribution statement

**José Carlos Paiva:** Conceptualization, Methodology, Software, Validation, Formal analysis, Investigation, Resources, Data curation, Writing – original draft, Writing – review & editing, Visualization, Project administration, Funding acquisition. **José Paulo Leal:** Conceptualization, Methodology, Validation, Resources, Writing – review & editing, Supervision, Project administration, Funding acquisition. **Álvaro Figueira:** Conceptualization, Methodology, Validation, Resources, Writing – review & editing, Supervision, Project administration, Funding acquisition.

## Declaration of Competing Interest

The authors declare that they have no known competing financial interests or personal relationships that could have appeared to influence the work reported in this paper.

## Data Availability

PROGpedia (Original data) (Zenodo).
